# Women’s Health and Well-Being in the United Nations Sustainable Development Goals: A Narrative Review of Achievements and Gaps in the Gulf States

**DOI:** 10.3390/ijerph17031059

**Published:** 2020-02-07

**Authors:** Suhad Daher-Nashif, Hiba Bawadi

**Affiliations:** 1 Population Medicine Department, College of Medicine, QU-health, Qatar University, Doha 2713, Qatar; snashif@qu.edu.qa; 2 Human Nutrition Department, College of Health Sciences, QU-health, Qatar University, Doha 2713, Qatar

**Keywords:** women, well-being, maternal mortality, sexual health, Gulf states, SDG 3

## Abstract

*Background:* In 2014, United Nations member states proposed a set of sustainable development goals (SDGs) to help further the millennium development goals that they had proposed in New York in 2000. Of these 13 SDGs, Goal 3 (i.e., SDG 3) was titled “Good Health and Well-Being.” This goal highlighted women’s health and well-being via two key objectives. The first, SDG 3.1, aimed to reduce maternal mortality rates (MMR) and the second, SDG 3.7, aimed to ensure access to sexual and reproductive health care services. Drawing on all the latest reports, which have been released by Gulf Cooperation Council states (GCC), this paper sheds light on GCC states’ work on women’s wellbeing through SDG 3. *Aim:* the paper aims to review GCC states’ work on women’s wellbeing in SDG3, which achievements they obtained, which tools they used and which gaps still exist. The paper aims to explain the socio-cultural background behind these achievements, tools, and gaps. *Methodology:* For the purpose of this study, we used narrative review approach through which we reviewed reports from 2017 and 2018 on SDGs published online by the Ministry of Development and Planning of each GCC state, and latest reports of the WHO on the same states. *Findings:* the study found similarities and differences between different GCC states, which in turn reveals gaps and areas that are not meeting women’s needs. The findings show that MMR in GCC countries has declined by nearly half. The main strategies they adopted to address SDG 3.1 included awareness campaigns, improving access to healthcare systems and training professionals. The tools used to meet SDG 3.7 included training health professionals and raising awareness of consanguinity. The study reveals several gaps, such as a lack of discussion around challenges and barriers, and a lack of linkage between an SDG and the targets contained within it. *Conclusion:* The paper concludes that there is a much greater emphasis on reducing MMR, compared to providing access to sexual and reproductive healthcare. This difference is due to different socio-cultural framing of each of these two issues.

## 1. Introduction

In 2000, the United Nations (UN) proposed eight millennium development goals (MDGs), which specifically sought to achieve certain milestones by 2015. Their aims included eradicating extreme poverty and hunger; achieving universal primary education; promoting gender equality and empowering women; reducing child mortality; improving maternal health; combatting the human immunodeficiency virus and its associated acquired immunodeficiency syndrome(HIV/AIDS), malaria, and other diseases; ensuring environmental sustainability; and developing a global partnership for development [1]. In 2015, the UN reinforced these MDGs with 17 sustainable development goals (SDGs), which represented the focus of the states’ agenda until 2030 [2]. These SDGs included objectives targeting economics, the environment, education, gender, and health, and were described by the UN Secretary-General Ban Ki-moon as a “shared vision of the humanity and social contract between the world’s leaders and the people” [1]. Each SDG contains a number of smaller targets and indicators, to measure achievement [3]. These SDGs came into effect in 2016, so UN member states have now started implementing the agenda for 2030 by creating a transformational action plan that is based on the 17 SDGs. 

Gulf Cooperation Council (GCC) countries (i.e., Bahrain, Kuwait, Oman, Qatar, the Kingdom of Saudi Arabia (KSA), and the United Arab Emirates (UAE) aligned their national development plans with these new goals and affirmed their engagement with and commitment to fulfilling these goals [4]. Each country devised its own plan, based on its own needs, available resources, and socio-cultural visions and ideologies. This paper sheds light on the ways in which Gulf states targeted women’s health and well-being, in terms of SDG 3 (specifically targets SDG 3.1 and SDG 3.7). The title of SDG 3 is “Good Health and Well-Being,” which seeks to ensure healthy lives and to promote well-being for all at all ages. Within this overarching aim are nine smaller targets, of which SDG 3.1 and SDG 3.7 directly focus on women’s health. SDG 3.1 endeavors, by 2030, to reduce the global maternal mortality ratio to less than 70 per 100,000 live births. Maternal mortality is defined as “the death of a woman during pregnancy, childbirth, or within 42 days postpartum” [5]. It is assumed that this goal emerged from the fact that the maternal mortality rate (MMR) decreased by 44% globally, from 2010 to 2014 [6]. SDG 3.7 aims, by 2030, to “ensure universal access to sexual and reproductive healthcare services, including family planning, information and education, and the integration of reproductive health into national strategies and programs” [7]. By reviewing the latest reports published by each GCC state’s Ministry of Development and Planning, this paper examines the ways in which these states targeted women’s health and well-being. This review enabled us to explore the socio-cultural attitudes and perceptions of women’s well-being, as well as the ways in which this is linked to other sociodemographic factors related to women.

Health and well-being were first defined by the WHO in 1948 as “a state of complete physical, mental, and social well-being, and not merely the absence of disease or infirmity” [8]. This definition constituted the basis for the structure of SDG 3, and it is used by various countries as a compass for their plans to reach SDG 3 targets. Thus, a question emerges about how this definition is reflected in the plans and performances of the GCC countries. In order to answer this question, this article examines the achievements of these states, in terms of targets SDG 3.1 and SDG 3.7, as well as the main strategies and tools that were employed to achieve these targets. 

In this article, GCC states constitute a case study of a Middle Eastern context, one in which socio-cultural factors affect preferences about implementing the UN’s SDGs, and affect the tools used to achieve these targets and goals. The paper examines how the global discourse, which is mostly western, is implemented, paraphrased, and re-shaped in Middle Eastern contexts. This is an invitation to re-examine the unified discourse in a multi-cultural world. 

## 2. Methodology

In order to uncover achievements, tools, and gaps in targeting women’s health and well-being in GCC states, this study uses narrative review methodology. The study reviews the last (2017, 2018) reports on SDGs published online by the Ministry of Development and Planning of each GCC state, and the latest reports of the WHO on the same states. Narrative review is the appropriate tool to document and analyze the reports, because it is one of the review research methodologies through which existing studies are summarized (reports in our case), from which conclusions may be drawn into a holistic interpretation contributed by the reviewers [9]. Narrative review studies’ results are qualitative rather than quantitative, and enable the researcher to acknowledge, reflect, and assist the reviewed data [10]. All reports were first accessed during December 2018. This narrative review focuses on three main themes: achievements, the tools and strategies that were employed, and gaps that remained in the reports. The following section will discuss these three themes, with regards to targets SDG 3.1 and SDG 3.7.

## 3. Findings

### 3.1. SDG 3.1: Reducing the Global Maternal Mortality Ratio

This target includes two indicators: MMR and skilled birth attendance. The latest reports from the GCC states mainly focus on the decrease in the MMR and on the ways in which they reduced it, but less emphasis is placed on skilled birth attendance. However, in some reports, skilled birth attendance is a strategy employed to reduce MMR. For example, although several studies [11] illustrate that skilled staff can reduce maternal mortality, only reports from Oman [12], Qatar [13], and the KSA [14] explicitly mention that certifying skilled staff was one of the tools used to reduce the MMR [15]. It is important to note here, that the WHO’s 2015 reports on the MDGs, indicates that all GCC countries reduced the MMR by roughly half between 1999 and 2015 as indicated in Table 1. For example, Bahrain’s MMR decreased from 26 to 13, the KSA’s decreased from 46 to 17, and Qatar’s decreased from 29 in 1990 to 4 in 2015 [16]. The reviewed reports highlight the work of each state on reducing the MMR. For example, the UAE report highlights the fact that the current MMR is 6 per 100,000 live births, and the target is to reach 3 per 100,000 by 2030. They used awareness campaigns to encourage breastfeeding as a preventive tool. This downward trend in the GCC aligns with a global reduction in MMR; one meta-analysis study, which was conducted over a 28 year period (1980–2008), reveals a downward trend in MMR in 181 countries [5]. This study attributes this trend to improvements in services and public awareness, but also notes that it is likely the result of an improvement in human development index (HDI) components, such as life expectancy, education, and income [17]. Rahman et al. [16], for instance, report a correlation between the high level of female literacy in Qatar from 1974 to 1993 with the decline in child and maternal mortality rates.

In their reports, each GCC outlines the tools they used to reduce the MMR. These tools can be categorized into three main themes: prevention, treatment, and re-structuring the approach to healthcare. Preventive tools included awareness campaigns and events aimed at women in all GCC states and kingdoms, while some states also targeted young people (Kuwait) [20] and men (Qatar) [13]. In addition to awareness, Bahrain [21] and Qatar’s preventive tools included improving access to healthcare systems.

Treatment tools included improving the quality of service by enforcing high standards of treatment (Bahrain, Qatar, and the KSA), training professionals (Oman, Qatar, and the KSA), and direct supervision of and advice for women (the KSA, Oman, and the UAE [22]). A patient-centered approach is one of the methods repeatedly mentioned and used by multiple GCC states. For example, Oman, Qatar, the KSA, and the UAE all report employing patient-centered approaches as both preventive and treatment tools. Collaboration between the private and the public sectors was an additional approach employed to re-structure healthcare services (Bahrain, Qatar, and the KSA). However, some tools and approaches were specific to a certain state. For example, Qatar’s focus on human rights and socio-psychological care. The KSA note the importance of referring to Sharia ethics during patient care, while the UAE employed the body-friendly concept. It is noteworthy that, although these reports reveal many similarities in states’ strategies for lowering the MMR and although the socio-cultural contexts of these states are very similar, none of the reports mentioned collaborating with another GCC state on a shared approach or strategy.

The second target, SDG 3.7, directly focuses on women’s health and well-being. The following section outlines GCC states’ work towards this target.

### 3.2. SDG 3.7: Ensuring Access to Sexual and Reproductive Health Care Services

This target includes three indicators: total fertility rate, contraceptive prevalence rate, and meeting the demand for family planning. In the reports released by GCC states and kingdoms, only Bahrain, Oman, Qatar, and the UAE describe their work towards achieving this target. Bahrain, for example, outlines its efforts towards decreasing the adolescent (15–19) birth rate, as a tool for the prevention of MMR, which reveals that the government links early marriage and pregnancy to MMR. The Omani report details their work on family planning [12]:

In the latest data available (for 2014), the rate of contraceptive use in Oman is still low. The percentage of women of reproductive age (15–49 years) using modern methods of birth spacing in Oman was 18.8%, and 29.7% for those using both traditional and modern methods together. The adolescent birth rate in the Sultanate has recorded a slight increase in the last two years, reaching 13.5 per 1000 adolescent women in 2016 [12]

The report also describes the tools utilized to reach this target, such as training healthcare professionals to provide life-saving obstetric care, including providing the necessary supervision, care, and advice to women during pregnancy, labor, and in the postpartum period. The Qatari report comments on work to raise awareness of the dangers of cousin marriages and consanguinity, while the UAE’s report explains that their main tool was providing immediate access to the information needed for planning and decision-making.

### 3.3. Gaps in Reporting SDG 3.1 and SDG 3.7

The reports place a special focus on maternal and child death, while marginalizing the issue of sexual and reproductive health rights. Some researchers have argued that the reproductive health needs of young Arab people are not fully met, due to a societal reluctance to address these issues, as well as cultural and religious sensitivities [23,24]. These sensitivities generally intensify when the focus is on women’s needs. This may explain why the focus on target SDG 3.1 (related to MMR) greatly exceeds the focus on target SDG 3.7 (related to access to sexual and reproductive healthcare).

A report issued by the Middle East and North Africa Health Policy Forum (MENA-HPF) [25] studied Saudi Arabia as one of six countries deemed representative of the Gulf states. The MENA-HPF report asserted that “reproductive health services are still not comprehensive in the kingdom; they consist mainly of maternal and child health, with very few sexual health services” [25]. In another report released by the same forum in 2016 [26], Saudi Arabia and the UAE were among 11 MENA countries that had reviewed these laws and policies. The report states that, although the selected countries have had many achievements, many issues related to sexual and reproductive health remain relatively neglected. This may explain the absence of these issues in most of the SDG 3 reports examined in this paper. The MENA-HPF report also notes that “all countries under review report the existence of national strategies on population, youth, women, and reproductive health, but the human rights-based approach is not well represented in those strategies” [25]. The report also mentions that the KSA and UAE were among the countries encouraging their citizens to conduct premarital screening for genetic and infectious diseases. This raises the question of what is suggested for women in cases where a fetus is found to have a physical defect, including the type of support that a mother might be given. Both the KSA and UAE were taken as representative GCC states, but the report also notes that in most of the GCC states laws protect women from human trafficking for sexual exploitation. It also reports that there is no legal standard for marriageable age in the KSA or UAE, which leaves young women and girls vulnerable to social practices and traditional laws, like forced marriage.

### 3.4. Information Lacking in the Reports

In general, the reports lack discussion of any obstacles and challenges that arose during these years of work within the SDG framework, both in terms of health in general and women’s health in particular. Moreover, socio-cultural, environmental, legal, and political challenges are absent from all the reports. Saleh and Taleb [27] define the barriers to the integration of sustainability practices within the GCC, including difficulties in achieving client buy-ins to sustainable construction, limited public awareness, heavily subsidized prices for electricity and water, and limited knowledge and/or misinformed views among professionals about the notion of sustainability. Hogan et al. [5] argue that income per capita can reduce maternal mortality through several channels. Although the GCC’s economic status has enabled the implementation of all improvements and new construction in many domains, the reports did not discuss plans to preserve sustainability in the case of unexpected events, such as natural disasters, wars, or economic crises.

These reports also lack documentation of laws and regulations that were issued as part of their plans to achieve SDG 3. Furthermore, the reports lack a holistic approach, i.e., no links are made between SDG 3 and other SDGs. For example, none of the reports describe using environmental regulations as a tool to enhance the health and well-being of pregnant women and newborn babies. According to Bosselmann [28], legislation on ecological sustainability is a key factor to achieve health sustainability. Therefore, this claim raises further questions about the ways in which GCC states have used one goal to achieve targets in another goal.

Furthermore, connections are not made between targets within the same SDG. For example, there is no information on how states might address the psychological well-being of a mother after losing a child, or on actions taken regarding women’s psychological and physical health as a means of preventing maternal and childhood mortality. In general, the issue of women’s mental health is marginalized in these reports, despite being a critical part of the WHO’s definition of health.

The reports also discuss the same strategies for young and old women, without differentiation between adolescence and adulthood. This generalization suggests that these states define women as one, homogenous category, without a deeper understanding of the different health and well-being needs of women of different ages. Culture-related issues are similarly neglected, as differing needs among different cultures living within these states are also ignored.

## 4. Discussion and Recommendations

GCC states and kingdoms should utilize the potential for regional cooperation and economic integration, which would allow them to speak with a shared voice. This would also facilitate the states learning from each other and supporting each other’s implementation of plans to achieve SDG 3, as well as all SDGs. At the UN Development Group’s Arab Development Forum, held in Amman in 2013, regional cooperation was identified as a key driver of development in Arab countries [29]. This meeting also highlighted five values of particular importance to the Arab region: equity, resilience, sustainability, accountability, and participation.

The reports should focus on development processes, more than on the outcomes. For example, the reports lack any information about the ways in which states combined the universal framework with nationally relevant targets. This is a key conceptual issue that should be considered by GCC stakeholders, in order to ensure that global outcomes are also adequate at the local level. The MDGs and SDGs can be considered global in nature, but not universally applicable to all countries; that is to say, they are not tailored to regional and national contexts, and do not acknowledge national priorities. This may explain why some targets were given more attention than others in the GCC reports.

The last issue that should be taken into consideration, when working towards and reporting on SDG 3, is links between SDGs and connections among targets within SDG 3 itself. The nine targets encompassed in SDG 3 broadly fall into separate but overlapping groups. These are reducing morbidity and mortality among vulnerable groups (mothers, newborns, the elderly, and children), reducing communicable and non-communicable diseases, reducing risk factors (tobacco, substance abuse, road traffic injuries, and hazardous chemicals and pollution), providing universal health coverage, and strengthening the health sector [30]. Achieving one target can also help achieve another. For example, satisfying the target of reducing non-communicable and communicable diseases (SDG 3.3) could also assist a reduction in maternal mortality (SDG 3.1). This can be implemented at the policy level by, for example, addressing the prevalence of non-communicable diseases, such as diabetes, or mitigating risk factors, such as obesity and smoking, during pregnancy. 

Achieving health and well-being for all, including women, not only depends on achieving SDG 3, but also on other SDGs, such as ending poverty (SDG 1), improving access to education (SDG 4), and guaranteeing gender equity (SDG 5), among others [31]. Health and well-being also depend on sufficient services and resources, such as food security and agricultural production (SDG 2), access to energy (SDG 7), and sustainable consumption (SDG 12) [31]. Furthermore, there is now recognition of the broad interdependence between environmental and human health, as systems thinking now focuses on planetary health [32,33]. These connections may be implicitly acknowledged by GCC states and kingdoms, but they are not made explicit in their reports.

Lastly, SDG 3.7 will assist in the reduction of target SDG 3.1, and will also help control communicable disease (SDG 3.3). States can also meet target SDG 3.7 by funding reproductive and sexual health services and education, as well as by developing a strong health workforce and maintaining a supportive research infrastructure. Although research infrastructure is one of the strengths of several GCC states, evidence-based planning is missing from the reports. As such, this issue should be addressed in future planning, performance, and reporting.

## 5. Conclusions

This paper examined how the GCC states implemented UN’s SDG 3.1 and SDG 3.7 which focus on women’s health within their societies. Based on GCC states’ reports, the study found that the work on MMR was stronger than the work on access to sexual and reproductive health care services. This difference can be referred to the socio-cultural values, attitudes, meanings and perceptions of each of these two issues. The reports indicate the lack of collective vision for wellbeing. UN discourse surrounding health and wellbeing should be international that considers social and political determinants of health. 

## Figures and Tables

**Table 1:** Maternal mortality rates (MMR) (deaths per 100,000 live births) in Gulf Cooperation Council (GCC) countries from 2000–2017 *.

Country	2000	2005	2010	2015	2017	% reduction in MMR (2000-2017)
Bahrain	27	19	18	15	14	48
Kuwait	10	10	10	11	12	-20
Oman	20	19	18	19	19	5
Qatar	14	12	10	9	9	36
Saudi Arabia	24	22	19	17	17	29
United Arab Emirates	6	5	4	3	3	50

Source: UN Maternal Mortality Estimation Group (World Health Organization, United Nations Children’s Fund, United Nations Population Fund, and World Bank) [18]; * The year 2015 marks the end of the millennium development goal (MDG) era and the beginning of the sustainable development goal (SDG) era. For the final evaluation in MDG 5 (which calls for a reduction of 75% in the MMR between 1990 and 2015), the United Nations Maternal Mortality Estimation Inter-agency Group (UN MMEIG) carried out a comprehensive assessment of MMR levels and trends for 183 countries [19].
